# Vulnerable but stronger together: An interpretative phenomenological analysis of the experiences of mothers of young adults with profound intellectual and multiple disabilities during the COVID-19 pandemic

**DOI:** 10.3109/13668250.2022.2135174

**Published:** 2022-11-08

**Authors:** M. C. den Boer, M. A. C. Voermans, P. J. C. M. Embregts

**Affiliations:** a Tranzo, Tilburg School of Social and Behavioral Sciences, Tilburg University, Tilburg, The Netherlands; b Amarant, Healthcare Organisation for People with Intellectual Disabilities, Tilburg, The Netherlands

**Keywords:** Profound intellectual and multiple disabilities, mothers, COVID-19, interpretative phenomenological analysis

## Abstract

**Background:**

Mothers of young adults with profound intellectual and multiple disabilities that live at home are less likely to be on the radar of formal services. We explored the experience of these mothers over the course of the COVID-19 pandemic.

**Methods:**

A qualitative study using three case studies. Transcripts were analysed using Interpretative Phenomenological Analysis.

**Results:**

Two overarching themes with various subthemes emerged. The first theme focuses on the impact of being a mother of a young adult with profound intellectual and multiple disabilities who lives at home during the COVID-19 pandemic. The second theme describes both the vulnerability and resilience of the broader system, as well as its need to experience togetherness.

**Conclusions:**

Families of a young adult with profound intellectual and multiple disabilities exhibit both resilience and vulnerability during the COVID-19 pandemic, underscoring the importance to support and promote the visibility of these families.

Since its onset in late 2019, the COVID-19 pandemic has profoundly impacted upon all segments of society. People with intellectual disabilities are particularly vulnerable to the physical, mental, and social effects of the pandemic (Courtenay & Perera, 2020; Doody & Keenan, 2021). Indeed, several studies have demonstrated how the pandemic has negatively impacted upon the physical health of people with intellectual disabilities, both in terms of being at a higher risk of contracting COVID-19 and having a higher mortality rate from it (Gleason et al., 2021). Furthermore, people with intellectual disabilities are at an increased risk of mental illness, loneliness, agitation, anxiety, and distress (Doody & Keenan, 2021). Apart from the negative impact of governmental measures on people with intellectual disabilities (e.g., social isolation), these measures can furthermore be difficult to understand, which in turn can also affect their mental health (Courtenay & Perera, 2020).

Those with profound intellectual and multiple disabilities have especially high prevalence rates of health problems associated with poorer COVID-outcomes, such as heart disease or respiratory disease (van Timmeren et al., 2017). Due to their profound intellectual disabilities, severe neuromotor dysfunction, and attendant medical problems, people with profound intellectual and multiple disabilities depend on help from family members and professional support staff (Kruithof et al., 2020). In the Netherlands, care and support for people with profound intellectual and multiple disabilities is generally provided within a professional care setting, with only a minority living in their family home (Vugteveen et al., 2014). Even when living in their family home, people with profound intellectual and multiple disabilities rely on professional services, such as day activity centres and respite care (Luijkx et al., 2017). However, as a result of the COVID-19 measures, these facilities were often closed or operating at reduced capacity (Boeije et al., 2021; Doyle, 2021).

Closing professional services undoubtedly affected parents, especially those whose children lived with them, as they were unable to receive help with care (Courtenay & Perera, 2020). Parents of individuals with profound intellectual and multiple disabilities living at home are less likely to be on the radar of formal services (Kruithof et al., 2021). There is scarce knowledge about the pandemic’s impact upon this group of parents. Various studies have reported the experiences of parents with a child with an intellectual disability living at home. Generally, parents reported experiencing greater stress, pressure, and a deterioration in their mental health or quality of life (Paulauskaite et al., 2021), substantial and serious levels of worry (Asbury et al., 2021; ten Brug et al., 2021) or even significantly higher levels of anxiety and depression during the first lockdown in comparison to carers of children without intellectual disabilities (Willner et al., 2020). Although some studies included parents with a child with profound intellectual and multiple disabilities in their sample (Embregts et al., 2021; Kim et al., 2021; Patel et al., 2021), none of them aimed to provide specific insight into this group’s experiences, and most solely focused on the first lockdown or the initial stages of the pandemic. However, although restrictions were eased after the first lockdown, various restrictions remained in place long after this first lockdown, and, in fact, various countries introduced new lockdowns (European Commission, Joint Research Centre, 2021). Neece et al. (2020) reported that parents of individuals with intellectual disabilities expressed concerns over whether the measures would continue for an extended period of time, including concerns over finances, emotional concerns for themselves and their children, and concerns about the lack of educational and developmental progress of their child. However, an in-depth understanding of the impact of the COVID-19 pandemic upon parents of individuals with profound intellectual and multiple disabilities as well as the difficulties they may face as a consequence of the long-lasting pandemic is lacking.

To gain insight into how the COVID-19 pandemic has impacted upon these parents, we studied the experiences of parents of young adults with profound intellectual and multiple disabilities who live at home during the COVID-19 pandemic. To gain an in-depth understanding of their experiences, we conducted an Interpretative Phenomenological Analysis (IPA) (Smith et al., 2009) on a small sample, using three case studies. This study aimed to explore how mothers of young adults (16–30 years) with profound intellectual and multiple disabilities living at home experienced the pandemic, how the pandemic affected their wellbeing, that of their young adults with profound intellectual and multiple disabilities and their families, and how they and their families coped during different phases of the pandemic.

## Method

### Participants

The participants were identified, informed, and recruited to take part in the study by a contact person in healthcare organisations affiliated with the Academic Collaborative Centre Living with an Intellectual Disability (Tilburg School of Social and Behavioral Sciences, Tilburg University, the Netherlands). In line with principles of IPA, participants were purposively sampled in order to present a homogeneous sample (Pietkiewicz & Smith, 2014). Mothers needed to have a young adult with profound intellectual and multiple disabilities who (I) lived at home prior to the pandemic, (II) was living at home during the pandemic, and (III) was aged 16–30 years. Three mothers, aged 47–57 years (average 50.33 years), participated in this study. Demographic details and information about both the mothers and their young adults’ family life were obtained during the first interview and are shown in Table 1, using pseudonyms to ensure anonymity. All mothers had a daughter with profound intellectual and multiple disabilities (reported as such by mothers themselves) that required assistance with all personal care. Furthermore, all mothers were the breadwinner in their families. At the time of interviews, Emma and Iris were mostly working from home, yet, when governmental measures permitted, they were sometimes working in-office too. Nina, who started her own catering company, prepared meals at home and delivered them at her costumers’ place, meanwhile preventing physical contact with her customers. Emma and Iris lived in nuclear families, while Nina was divorced and living with a new partner and two of her four children. Prior to the pandemic, all mothers used professional services for their daughters with profound intellectual and multiple disabilities. After the closure of professional services during the first lockdown, Emma and Iris began to use them again during the Summer of 2020, whereas Nina decided not to use professional services during the period under consideration, as she was concerned about the risk of her daughter getting infected with the coronavirus.

**Table 1.** Demographic information of participating mothers and their child with profound intellectual and multiple disabilities (PIMD).

Mother’s name	Mother’s age	Type of family	Number of children	Number of children that lived at home during the pandemic	Name of child with PIMD	Age of child with PIMD	Level of functioning	Additional diagnosis	Type of professional services prior to the COVID-19 pandemic	Type of professional services during last interview
Nina	57	Divorced, lives together with a new partner	4	2	Meghan	26	4 months	Epilepsia, one lung, blind	2 days at day-care centre; 3 days activities at home; in-home care	None
Emma	47	Nuclear	1	1	Daisy	17	1.5 years	Cerebral palsy, epilepsia	5 days at day-care centre	5 days at day-care centre
Iris	47	Nuclear	3	3	Johanna	19	6 months	Epilepsia, metabolic disease, rare genetic disease	5 days at day-care centre; in-home care	5 days at day-care centre; in-home care

### Study design

An IPA framework was used in this study (Smith et al., 2009). Data collection via semi-structured interviews took place between 23rd November 2020 and 3rd May 2021. From 14th October to 15th December 2020, a partial lockdown was in place in the Netherlands, which included restrictions in daily visitors and group sizes, closure of establishments serving food and drinks, and mandatory wearing of masks in indoor places and on public transport. From 16th December 2020 to 28th April 2021, a stricter lockdown was introduced, which included the closure of all non-essential shops, schools, and indoor sporting venues. Furthermore, between 23rd January and 28th April, a night-time curfew was in force. From 28th April onwards, restrictions gradually lifted. As opposed to the first lockdown in March 2020, professional services generally remained open during the entire period after the first lockdown. However, professional services could be temporarily closed in case of shortage of staff due to infections or quarantine. Furthermore, various preventive measures were in place, e.g., usage of face masks, or limited or no possibilities for handover at the start and the end of a day spent at a day care centre.

Mothers were interviewed two or three times during the study. The first interview with Nina and Emma took place during the partial lockdown, while the first interview with Iris occurred during the first weeks of the strict lockdown. Due to the unforeseen strict lockdown, Nina and Emma were interviewed again at the beginning of the strict lockdown to also gain insight into their experiences of this period. The final interview took place in the final stage of the strict lockdown, that is, April/beginning of May 2021.

### Procedure

Ethical approval for the study was obtained from the Ethics Review Board of Tilburg University (RP149). With their consent, the first author contacted potential participants by phone to check the inclusion criteria and to further inform them about the study. If the mothers expressed interest in participating, then an information letter and informed consent form was sent to them via email along with an appointment for an interview.

Using an interview guide following IPA guidelines (Smith et al., 2009), semi-structured interviews were conducted. Interviews began as follows: “How are you at this stage of the pandemic?”. A dialogue with the mothers was established by using multiple open-ended questions, including “How does daily life look like?”, “How do you feel about the current situation?” and “Who or what keeps you going?”. The interview guide was used to ensure that the key topics were discussed, while still remaining open to other issues being raised by the participants. During the first interview, mothers were specifically asked about their experiences during the first stages of the pandemic. The interview guide for this interview was based on an earlier study, interviewing mothers caring for children with intellectual disabilities during the initial stage of the pandemic (Embregts et al., 2021). In subsequent interviews, mothers were asked about their experiences during the current stage of the pandemic. A similar interview guide was used for these interviews, albeit supplemented with topics relevant to the current stage of the pandemic (e.g., implementation of new measures or the vaccination program). The interview guide can be requested from the corresponding author.

Interviews were conducted by the first author. Due to governmental restrictions, interviews were conducted via video calls using Microsoft Teams. Interviews were recorded using the record function on this program and transcribed verbatim. Due to an unstable internet connection, one interview was conducted via telephone and audio recorded. Interviews lasted on average 47.2 (range 26–65) minutes.

### Analysis

Throughout the analysis, the stages described in the IPA guidelines (Smith & Shinebourne, 2012) were carefully applied. The analysis was carried out via close cooperation between two researchers (MB and MV). Furthermore, a careful audit process involving the entire research team (MB, MV, and PE) was built into each stage of the analysis. After reading and re-reading the transcripts, descriptive, linguistic, and conceptual comments were made, which were carefully audited. From these initial comments, emergent themes were developed and audited. In a subsequent meeting between two researchers, connections across themes were identified. These steps were repeated for each respondent for all interviews. Next, connections across themes within one case were identified and carefully audited. Finally, connections and patterns across cases were identified by the entire research team. All decisions made during the analysis were documented in a reflective journal.

## Results

Two overarching themes with various subthemes emerged from the analysis. The first theme, “My daughter’s health and a good life are my main priorities”, focuses on the impact of being a mother of a young adult with profound intellectual and multiple disabilities who is living at home during the COVID-19 pandemic. The second theme, “Vulnerable but stronger together: my need for a sense of togetherness in the larger system to which I, my daughter and my family belong,” describes both the vulnerability and strength of the broader systems that these mothers and daughters belong to and the importance of togetherness for this system to remain resolute.

### My daughter’s health and a good life are my main priorities

All of the mothers expressed that their daughters’ wellbeing was their main priority during the pandemic. This is addressed in the following subthemes.

#### Living in constant fear for my daughter’s health

All mothers reflected on their daughters’ vulnerability and expressed fear that they would get infected and become seriously ill, which appeared to be rooted in their daughters’ earlier experiences or medical history. For example, during an earlier period of serious illness, Meghan was initially refused critical care as physicians deemed that Meghan lacked perspective and quality of life. Therefore, Nina feared that Meghan would again be refused critical care if she became seriously ill from the coronavirus, and thus she was determined to prevent Meghan from getting infected.

Over time, the fear that their daughters would become infected reduced. For Emma, this stemmed from the fact that her family all coped surprisingly well after contracting the coronavirus. Although Nina and Iris found some effective coping strategies to deal with their fear, they are eagerly awaiting their families to get vaccinated. However, despite their frailty, neither people with profound intellectual and multiple disabilities (PIMD) who are living at home nor their family carers are prioritised for vaccination in the Netherlands. Both Nina and Iris thus feel unseen by the government and society:

The strange thing is that Johanna is not registered that way. True, she is vulnerable, but she lives at home. So she doesn't live within the walls of the institution, and if she did then she would be registered as vulnerable and she would also be relatively high up the [vaccination] schedule. But now she's just another 18–65-year-old living at home. And so she is put in that scheme. (Iris)

However, Nina and Iris do not expect their fear to vanish once either everybody is vaccinated or the pandemic ends. Rather, they noted that they will continue to approach daily social situations, such as visiting a shop, differently and with greater caution than they did before.

#### Ensuring a meaningful and safe life for my daughter: Never free from experiencing feelings of guilt

Throughout the pandemic, the mothers strove to ensure a safe and meaningful life for their daughters. However, concerns over their daughters’ quality of life grew the longer the restrictions went on. For example, the lack of meaningful activities and social contact caused Johanna to experience depressed feelings, which, in turn, led Iris to worry about her quality of life. Indeed, all the mothers recognised the importance of meaningful activities for their daughters’ quality of life, which is why they sought to ensure some continuation of these activities. In some cases, mothers offered these activities themselves, although they stressed that this was very demanding for them. Emma, for instance, spoke of how she struggled to balance work with her efforts to provide her daughter with meaningful activities:

So you constantly feel like you're not doing enough. You want to be with your daughter to keep her busy, to stimulate her, but you also have to work and I found that combination very difficult. Yes. There was a constant feeling of guilt, the feeling that you’re always doing too little in both respects. In terms of work and in terms of caring for your daughter. (Emma)

Some measures were lifted after the first lockdown, which presented greater opportunities to provide meaningful activities for their daughters, but also posed dilemmas for the mothers, insofar as they constantly had to balance their daughters’ quality of life with the risk of getting infected. Regardless of what choice the mothers made, it was evident that either could result in feelings of guilt. For example, despite the increased risk of infection, Emma decided to let Daisy go to the day activity centre again, because she knew this would improve her wellbeing. However, Emma felt strong guilt after her whole family got infected via the day activity centre. Conversely, Nina had decided not to restart professional services for her daughter, which also resulted in feelings of having deprived Meghan.

#### Revaluing a good life: Meaningful in the eyes of my daughter

All of the mothers spent more time with their daughters as a result of the governmental measures. This literal proximity to them enabled the mothers to better put themselves in their daughters’ shoes, thus resulting in new insights into what their daughters perceived as being a good life. For example, Nina noticed that Meghan really enjoyed simple activities with her sister:

And now I notice that she just really enjoys watching television with [her sister] for example (…) Then I think: well, that's another activity, yes, that's fine too. (…) I always used to think that sitting in front of the television was a bit, well, inferior. (Nina)

Iris also gained valuable insights into what was important for her daughter. During the initial lockdown, Johanna truly enjoyed spending more time together as a family. However, Johanna soon experienced depressed feelings, which led Iris to recognise the importance of social contact beyond the family for Johanna. Thereafter, she vowed to ensure that Johanna would get the contact she needs, both during the pandemic and after.

#### My daughter’s health is central, but who am I and what do I need?

Within a long-term severely restricted social context with increased demands and responsibilities, the mothers also began to reflect upon who they are and what they need for themselves. For instance, Emma expressed that she tries hard to balance her professional and personal life. However, she became aware that her own personal needs were not completely being fulfilled in the long-term severely restricted social context of her family:

Of course you are with your family. Yes, and I really like to be able to do things outside of my family as well. I need that too. Yes, to be in balance. (Emma)

While contact with people outside her family circle was also valued by Iris, the pandemic caused Nina to critically assess her own forms of social contact, which made her realise that many of her contacts are not so valuable and that she could in fact live without them.

### Vulnerable but stronger together: My need for a sense of togetherness in the larger system to which I, my daughter and my family belong

Initially, all the mothers were focused on protecting their daughters’ safety and wellbeing. Over time, mothers increasingly reflected on themselves but also on the larger systems they belong to as well as the importance of a sense of togetherness within this larger system, especially within their family and in sharing care with professionals. This is addressed in two subthemes.

#### A sense of togetherness within my family: We’re all making sacrifices, but together we’re strong

Initially, everything was centred around protecting their daughters with profound intellectual and multiple disabilities, which required notable sacrifices from the mothers and other family members, which were not questioned at that time. However, new situations arose when the measures were lifted. For example, whereas Johanna at first suffered mostly from social isolation, over time she became the one who spent the most time away from home, while her family were still restricted. This made Iris question the sacrifices her family had to make for Johanna.

Maybe I find it even harder for them, especially the middle one [daughter], (…) her social life is pretty much gone, I think. Yes, she has a really tough time and I might even look at that a little more, you know. Johanna, she does go to day care, she does sleep over (…) she does have a kind of life, if you know what I mean. She can just go ahead and do things. So yes, that kind of thing, that’s something we weigh up a bit. (Iris)

At the same time, there appeared to be a strong sense of togetherness in all the families. Indeed, the mothers noted that their strong family bonds helped them to cope during the pandemic. Moreover, the mothers expressed that the resilience they had built up previously by virtue of being a family with a child with profound intellectual and multiple disabilities helped them cope with the pandemic. Iris, for example, stressed that the pandemic re-emphasised to her that her family was her safe place, and that leniency, creativity, flexibility, resilience, and responsibility were her family’s most notable attributes that they had developed through their family history. Emma also reported resilience as a key strength of her family, which helped them to cope with the uncertainty of the pandemic.

#### Sharing care with professionals: The importance of a sense of togetherness

All the mothers reflected on the role played by professional services, both in terms of care and providing meaningful activities for their daughters. For example, due to the measures in the first lockdown, Nina was forced to care for Meghan herself without any professional support for the first time. Although this made her appreciate caring for Meghan herself, she stated that she is looking forward to sharing this care with professionals in the future. Emma felt that, prior to the pandemic, she and her husband had mostly cared for Daisy, but the forced closure of the day activity centre led Emma to realise that they shared the care with the professionals at the centre more than she initially thought. Consequently, she became cognisant of the fact that sharing care for Daisy with the day activity centre was actually crucial for maintaining the balance of her family. Iris had always felt that establishing a sense of togetherness in collaboration with the professionals who cared for Johanna was of critical importance. Before the pandemic, Iris managed to establish this sense of togetherness. When Iris restarted professional services for her daughter after the first lockdown, she noticed that the longevity of the pandemic and the measures had resulted in overburdened professionals and some forced changes in care. Therefore, she felt a reduced sense of togetherness with the professional support staff and fears that this may be a lasting consequence of the pandemic:

Are we going to get back what we once had? You know, we were getting there, or had already succeeded to a large extent in sharing care. And yes, because that's gone now, I hope that won't become the norm again, that we won’t go back to the old intramural system, so to speak, versus the outside world. (Iris)

## Discussion

The present study explored the lived experiences of mothers of young adults with profound intellectual and multiple disabilities who were living at home during the COVID-19 pandemic, providing valuable insights into its impact upon these mothers and their families. Within the long-term severely restricted social context during the COVID-19 pandemic in the period of March 2020–May 2021, the mothers strove to ensure a meaningful life for their daughters with profound intellectual and multiple disabilities, while, simultaneously, protecting them from potential infection. This resulted in increased demands and responsibilities for mothers, which, in turn, caused real concern. However, the mothers also drew strength from positive experiences, such as enhanced proximity to their daughters, the strength of their families, in terms of resilience and responsibility, and a sense of togetherness, which helped them to cope with the pandemic.

In line with other COVID-19 related studies on parents of individuals with an intellectual disability (Embregts et al., 2021; Kim et al., 2021), the mothers in our study reported notable concern over their frail daughters getting infected and becoming seriously ill. Over time, they were able to cope with this fear and make it less intrusive by accepting or anticipating it. Furthermore, the start of the vaccination program in the Netherlands in early 2021 brought mothers some relief. Nevertheless, the mothers reported that their fears did not fully subside, and that it would likely become a way of life that impacts how they view the world around them. Consequently, these concerns may have long-term mental consequences. These results are concordant with various studies expressing concerns over the impact of the pandemic upon the mental health of parents of people with intellectual disabilities, and which highlighted the importance of providing support to these parents during the initial stage of the pandemic (Embregts et al., 2021; Kim et al., 2021; Paulauskaite et al., 2021; Willner et al., 2020). Our results emphasise that it remains critically important to support these parents for as long as the pandemic lasts.

Besides concerns, our study also highlights the abundant strength and resilience of these families. Much like Rogers et al.’s (2021) qualitative research, the mothers in our study reflected upon how their family’s resilience helped them develop effective strategies to cope with the difficulties of the (longevity of) the pandemic. A sense of togetherness within their families allowed them to remain resolute, albeit the long-lasting consequences of the pandemic challenged this togetherness within their families. The longevity of the pandemic requires family members to make sacrifices for the person with profound intellectual and multiple disabilities for extended periods of time, which, in turn, can profoundly impact upon the quality of life of family members. Therefore, it is not only important to support parents; rather, as pre-pandemic studies show, the entire family system must be supported to ensure a good quality of life for all its members (Hastings, 2016; Seligman & Darling, 2017). This points towards the importance of providing respite care to these families, and emphasises the fact that authorities should strive to ensure that these services remain accessible if and when future governmental measures are implemented.

Another factor that is of paramount importance for the quality of life experienced by families caring for people with intellectual disabilities is the sense of feeling included in society (Geuze & Goossensen, 2021). Many families have reported experiencing stigma during the beginning of the pandemic (Embregts et al., 2021; Rogers et al., 2021; Vereijken et al., 2021) as well as the fear that people with intellectual disabilities will be forgotten by the government when the pandemic unfolds (Silverman, 2020). The latter was reported by the mothers in our study, as neither people with profound intellectual and multiple disabilities living at home nor their family carers were prioritised for vaccination in the Netherlands, whereas people with profound intellectual and multiple disabilities living in residential care facilities were. This may exacerbate the feeling among parents caring for individuals with profound intellectual and multiple disabilities living at home that they are a “hidden group” (Doody, Markey, & Doody, 2013; Ryan, Taggart, Truesdale-Kennedy, & Slevin*,* 2014). The experiences reported in this study underscore that, as the COVID-19 pandemic carries on, it is vital to ensure that both people with profound intellectual and multiple disabilities living at home and their families will not remain hidden, either when it comes to the vaccine strategy for booster vaccines or when implementing further restrictions.

### Limitations

The results of the present study should be interpreted in light of several limitations. First, although Nina and Emma were interviewed three times, Iris could only be interviewed two times due to later enrolment in this study and the sudden and unforeseen implementation of the strict lockdown. Moreover, as the COVID-19 still endures, the study period spans only a part of the pandemic. However, as we asked mothers to retrospectively reflect on their previous experiences in the first interview, we captured the experiences of mothers over a large part of the pandemic (i.e., March 2020–May 2021), which provides us with valuable insights in the enduring impact of the pandemic on these mothers and their families. In addition, the sample size was relatively small due to the difficulties involved in conducting this type of research when mothers are experiencing such long-term care demands. However, Smith et al. (2009) posit that a sample size of three participants is actually methodologically appropriate, as a distinctive feature of IPA is its commitment to detailed analysis of a small sample size (Smith & Osborn, 2015). Still, further research with more participants is needed. Furthermore, although we purposively sampled mothers in order to present a homogeneous sample, there may have been slight differences between daughters that needs to be considered. For instance, at the times of interviews, Daisy was at the stage of transitioning from child day care to adult day care, which was experienced as an additional complicating factor in times of the COVID-19. Moreover, we did not specify care needs of daughters. Differences in care needs may as well have impacted experiences of mothers. Additionally, participants in this study were relatively affluent and highly educated, and, therefore, their experiences cannot be generalised to all parents caring for young adults with profound intellectual and multiple disabilities living at home. Therefore, future research could also focus on the perspectives of less affluent families or families from different ethnic backgrounds, along with focusing on other family members, such as fathers and siblings, as well as on carers of male young adults with profound intellectual and multiple disabilities or cares of young adults with different care needs.

### Conclusion

In closing, the present study provides valuable insights into the experiences of mothers of young adults with profound intellectual and multiple disabilities during the COVID-19 pandemic. Our study revealed that families of young adults with profound intellectual and multiple disabilities living at home have exhibited enormous strength and resilience during the crisis. However, as these families are subjected to many concerns, including concerns about their frail daughters and the quality of life of their families, these families remain vulnerable, thus underscoring the importance of continued efforts to both support and promote the visibility of this group.

## References

[CIT0001] Asbury, K., Fox, L., Deniz, E., Code, A., & Toseeb, U. (2021). How is COVID-19 affecting the mental health of children with special educational needs and disabilities and their families? *Journal of Autism and Developmental Disorders*, *51*(5), 1772–1780. 10.1007/s10803-020-04577-2 32737668 PMC7393330

[CIT0002] Boeije, H., van Schelven, F., & Verkaik, R. (2021). *Gevolgen van coronamaatregelen voor naasten van mensen met een verstandelijke beperking*. Netherlands Institute for Health Services Research.

[CIT0003] Courtenay, K., & Perera, B. (2020). COVID-19 and people with intellectual disability: Impacts of a pandemic. *Irish Journal of Psychological Medicine*, *37*(3), 231–236. 10.1017/ipm.2020.45 32404232 PMC7287305

[CIT0004] Doody, C., Markey, K., & Doody, O. (2013). The experiences of registered intellectual disability nurses caring for the older person with intellectual disability. *Journal of Clinical Nursing*, *22*(*7-8*), 1112–1123. 10.1111/jocn.12020 23134212

[CIT0005] Doody, O., & Keenan, P. M. (2021). The reported effects of the COVID-19 pandemic on people with intellectual disability and their carers: A scoping review. *Annuals of Medicine*, *53*(1), 786–804. 10.1080/07853890.2021.1922743 PMC8205046 34110258

[CIT0006] Doyle, L. (2021). All in this together?’ A commentary on the impact of COVID-19 on disability day services in Ireland. *Disability & Society*, *36*(9), 1538–1542. 10.1080/09687599.2021.1935215

[CIT0007] Embregts, P., Heerkens, L., Frielink, N., Giesbers, S., Vromans, L., & Jahoda, A. (2021). Experiences of mothers caring for a child with an intellectual disability during the COVID-19 pandemic in The Netherlands. *Journal of Intellectual Disability Research*, *65*(8), 760–771. 10.1111/jir.12859 34076326 PMC8242374

[CIT0008] European Commission, Joint Research Centre. (2021). Response Measures Database (RMD). European Commission, Joint Research Centre (JRC) [Dataset] PID: http://data.europa.eu/89h/b649cb36-e63d-41c9-a57c-6d6d06dbae3c

[CIT0009] Geuze, L., & Goossensen, A. (2021). Exploring the experiences of Dutch parents caring for children with profound intellectual and multiple disabilities: A thematic analysis of their blogs. *Global Qualitative Nursing Research*, *8*, 1–11. 10.1177/23333936211028170 PMC8252404 34263011

[CIT0010] Gleason, J., Ross, W., Fossi, A., Blonsky, H., Tobias, J., & Stephens, M. (2021). The devastating impact of Covid-19 on individuals with intellectual disabilities in the United States. *NEJM Catalyst Innovations in Care Delivery*, *2(2)*, 10.1056/CAT.21.0051

[CIT0011] Hastings, R. (2016). Do children with intellectual and developmental disabilities have a negative impact on other family members? The case for rejecting a negative narrative. *International Review of Research in Developmental Disabilities*, *50*, 165–194. 10.1016/bs.irrdd.2016.05.002

[CIT0012] Kim, M. A., Yi, J., Jung, S. M., Hwang, S., & Sung, J. (2021). A qualitative study on parents’ concerns about adult children with intellectual disabilities amid the COVID-19 pandemic in South Korea. *Journal of Applied Research in Intellectual Disabilities*, *34*(4), 1145–1155. 10.1111/jar.12875 33694235 PMC8237012

[CIT0013] Kruithof, K., Olsman, E., Nieuwenhuijse, A., & Willems, D. (2021). I hope i’ll outlive him”: A qualitative study of parents’ concerns about being outlived by their child with profound intellectual and multiple disabilities. *Journal of Intellectual & Developmental Disability*, *47*(2), 1–11. 10.3109/13668250.2021.1920377. 39818581

[CIT0014] Kruithof, K., Willems, D., van Etten-Jamaludin, F., & Olsman, E. (2020). Parents’ knowledge of their child with profound intellectual and multiple disabilities: An interpretative synthesis. *Journal of Applied Research in Intellectual Disabilities*, *33*(6), 1141–1150. 10.1111/jar.12740 32367663 PMC7687241

[CIT0015] Luijkx, J., Van der Putten, A., & Vlaskamp, C. (2017). Time use of parents raising children with severe or profound intellectual and multiple disabilities. *Child: Care, Health and Development*, *43*(4), 518–526. 10.1111/cch.12446 28156014

[CIT0016] Neece, C., McIntyre, L. L., & Fenning, R. (2020). Examining the impact of COVID-19 in ethnically diverse families with young children with intellectual and developmental disabilities. *Journal of Intellectual Disability Research*, *64(10)*, 739–749. 10.1111/jir.12769 32808424 PMC7461180

[CIT0017] Patel, V., Perez-Olivas, G., Kroese, B. S., Rogers, G., Rose, J., Murphy, G., Cooper, V., Langdon, P. E., Hiles, S., & Clifford, C. (2021). The experiences of carers of adults with intellectual disabilities during the first COVID-19 lockdown period. *Journal of Policy and Practice in Intellectual Disabilities*, *18*(4), 252–262. 10.1111/jppi.12382. PMC8242525 34226830

[CIT0018] Paulauskaite, L., Farris, O., Spencer, H. M., Group, E.-I., & Hassiotis, A. (2021). My son can’t socially distance or wear a mask: How families of preschool children with severe developmental delays and challenging behavior experienced the COVID-19 pandemic. *Journal of Mental Health Research in Intellectual Disabilities*, *14*(2), 225–236. 10.1080/19315864.2021.1874578

[CIT0019] Pietkiewicz, I., & Smith, J. A. (2014). A practical guide to using interpretative phenomenological analysis in qualitative research psychology. *Psychological Journal*, *20(1)*, 7–14.

[CIT0020] Rogers, G., Perez-Olivas, G., Stenfert Kroese, B., Patel, V., Murphy, G., Rose, J., Cooper, V., Langdon, P. E., Hiles, S., Clifford, C., & Willner, P. (2021). The experiences of mothers of children and young people with intellectual disabilities during the first COVID-19 lockdown period. *Journal of Applied Research in Intellectual Disabilities*, *34*(6), 1421–1430. 10.1111/jar.12884. 33759291 PMC8250127

[CIT0021] Ryan, A., Taggart, L., Truesdale-Kennedy, M., & Slevin, E. (2014). Issues in caregiving for older people with intellectual disabilities and their ageing family carers: A review and commentary. *International Journal of Older People Nursing*, *9*(*3*), 217–226. 10.1111/opn.2014.9.issue-3 23497304

[CIT0022] Seligman, M., & Darling, R. B. (2017). *Ordinary families, special children: A systems approach to childhood disability*. Guilford Publications.

[CIT0023] Silverman, A. (2020). People with intellectual disabilities may be denied lifesaving care under these plans as coronavirus spreads [Press release]. https://www.propublica.org/article/people-with-intellectual-disabilities-may-be-denied-lifesaving-care-under-these-plans-as-coronavirus-spreads

[CIT0024] Smith, J. A., Flowers, P., & Larkin, M. (2009). *Interpretative phenomenological analysis: Theory, method & research*. Sage.

[CIT0025] Smith, J. A., & Osborn, M. (2015). Interpretative phenomenological analysis as a useful methodology for research on the lived experience of pain. *Britsh Journal of Pain*, *9*(1), 41–42. 10.1177/2049463714541642 PMC4616994 26516556

[CIT0026] Smith, J. A., & Shinebourne, P. (2012). *Interpretative phenomenological analysis*. American Psychological Association.

[CIT0027] ten Brug, A., Luijkx, J., & Brander, A. (2021). Quarantine in a house of cards. The impact of The COVID-19 pandemic on the quality of life of relatives of people with major care needs. 10.21203/rs.3.rs-158520/v1

[CIT0028] van Timmeren, E. A., van der Schans, C. P., van der Putten, A. A. J., Krijnen, W. P., Steenbergen, H. A., van Schrojenstein Lantman-de Valk, H. M. J., & Waninge, A. (2017). Physical health issues in adults with severe or profound intellectual and motor disabilities: A systematic review of cross-sectional studies. *Journal of Intellectual Disability Research*, *61*(1), 30–49. 10.1111/jir.12296 27228900

[CIT0029] Vereijken, F. R., Giesbers, S. A. H., Jahoda, A., & Embregts, P. J. C. M. (2021). Homeward bound: Exploring the motives of mothers who brought their offspring with intellectual disabilities home from residential settings during the COVID-19 pandemic. *Journal of Applied Research in Intellectual Disabilities*, *35*(1), 150–159. 10.1111/jar.12930 34213037 PMC8420307

[CIT0030] Vugteveen, J., Putten, A. A. J., & Vlaskamp, C. (2014). *Inventarisatieonderzoek personen met ernstige meervoudige beperkingen: prevalentie en karakteristieken*. Stichting Kinderstudies & auteurs.

[CIT0031] Willner, P., Rose, J., Stenfert Kroese, B., Murphy, G. H., Langdon, P. E., Clifford, C., Hutchings, H., Watkins, A., Hiles, S., & Cooper, V. (2020). Effect of the COVID-19 pandemic on the mental health of carers of people with intellectual disabilities. *Journal of Applied Research in Intellectual Disabilities*, *33*(6), 1523–1533. 10.1111/jar.12811 32885897

